# The Struggle to Make CNS Axons Regenerate: Why Has It Been so Difficult?

**DOI:** 10.1007/s11064-019-02844-y

**Published:** 2019-08-06

**Authors:** James W. Fawcett

**Affiliations:** 1 John Van Geest Centre for Brain Repair, University of Cambridge, Robinson Way, Cambridge, CB2 0PY UK; 2 Centre of Reconstructive Neuroscience, Institute for Experimental Medicine ASCR, Prague, Czech Republic

**Keywords:** Axon regeneration, Chondroitin sulphate proteoglycans, NogoA, Schwann cell, Chondroitinase, Integrins, Signalling, Axonal transport, Trafficking, Rabs, RhoA, PTEN, Epigenetics

## Abstract

Axon regeneration in the CNS is inhibited by many extrinsic and intrinsic factors. Because these act in parallel, no single intervention has been sufficient to enable full regeneration of damaged axons in the adult mammalian CNS. In the external environment, NogoA and CSPGs are strongly inhibitory to the regeneration of adult axons. CNS neurons lose intrinsic regenerative ability as they mature: embryonic but not mature neurons can grow axons for long distances when transplanted into the adult CNS, and regeneration fails with maturity in in vitro axotomy models. The causes of this loss of regeneration include partitioning of neurons into axonal and dendritic fields with many growth-related molecules directed specifically to dendrites and excluded from axons, changes in axonal signalling due to changes in expression and localization of receptors and their ligands, changes in local translation of proteins in axons, and changes in cytoskeletal dynamics after injury. Also with neuronal maturation come epigenetic changes in neurons, with many of the transcription factor binding sites that drive axon growth-related genes becoming inaccessible. The overall aim for successful regeneration is to ensure that the right molecules are expressed after axotomy and to arrange for them to be transported to the right place in the neuron, including the damaged axon tip.

## History

Axon regeneration in the form of peripheral nerve repair has been in clinical practice since the 12th Century and possibly before, but spinal cord injury has been recognized as incurable since ancient times [14]. Cajal made several studies of regeneration in the PNS and its failure in the CNS, and was responsible with Tello for the first CNS regeneration experiment, implanting a graft of peripheral nerve into the CNS [106]. Yet we still do not have a solution for a clinically useful axon regeneration treatment. This stimulated a memorable introduction to a spinal injury research meeting by David Allan, director of the Scottish spinal injury unit, in which he challenged the audience,“the first regeneration experiment by Tello and Cajal was over 100 years ago, but we still do not have a treatment for our patients. What have you all been doing?”. Working out how to stimulate useful CNS axon regeneration has been extremely difficult, but recent progress has produced several interventions that enable partial regeneration, and increasing understanding of the biology gives reasonable optimism that a solution will be found soon. For many years research proceeded on the assumption that there might be a single straightforward fix for CNS regeneration. Peripheral nerves regenerate, and regeneration after axotomy in the CNS is generally successful in lower vertebrates and invertebrates, so surely the solution to lack of regeneration in the mammalian CNS should be straightforward. Sadly this is not so. It is now clear that evolution has gone to considerable lengths and developed several mechanisms to turn off regeneration and plasticity in the adult mammalian CNS. Restoring regeneration and plasticity is therefore complex, and several inhibitory mechanisms have to be addressed. Clearly there must be evolutionary advantages in turning off these developmental processes in adulthood. It may be that a hard-wired nervous system is more efficient, or the energetics or potential for mutation of maintaining regeneration and plasticity may be unfavourable, or there may be issues related to control of inflammation. Whatever the cause, we are now at the stage where the biology of regeneration failure is increasingly understood and solutions are being found. This review gives an overview of the current position.

## The Inhibitory CNS Environment

The fact that regeneration succeeds in peripheral nerves and fails in the CNS gives an obvious hypothesis that the PNS environment is permissive, the CNS is inhibitory. This idea was supported by Tello and Cajal’s grafting experiment, and further supported when Aguayo and his colleagues grafted peripheral nerves into the CNS and traced regenerating axons using modern methods [34, 43]. Clearly some CNS axons, particularly if their cell body is close to the graft, can regenerate axons into a permissive PNS environment. Grafting either nerves or permissive glia into the damaged spinal cord is therefore a method for enabling axon regeneration and for bridging a lesion cavity. Schwann cells have the advantage that they can readily be obtained from nerve explants from individual patients and expanded in tissue culture. Many animal experiments showed that spinal cord axons could regenerate into these grafts. However Schwann cells tend to be excluded from mixing with CNS glia by a reactive wall of astrocytes, and regenerating axons are inhibited at this point [1], so they rarely re-enter CNS tissue to re-connect to CNS neurons. This issue has been addressed by treating the lesion area with a phosphodiesterase inhibitor to raise cAMP [46, 60, 101], and by injection of chondroitinase ABC to digest the inhibitory CSPGs at the Schwan cell-astrocyte boundary. A clinical trial of autologous Schwann cell transplantation to human spinal cord injury patients is currently under way [3]. An alternative to Schwann cells are olfactory ensheathing glia (OEGs). The rationale is that the axons that constantly grow from newly born olfactory receptors are introduced into the CNS by these cells, which open the astrocyte boundary to enable them to enter the CNS environment. Transplantation in animal models led to successful regeneration and recovery [80], so a trial in human patients was initiated. Obtaining OEGs from human patients is more difficult than Schwann cells. Cells can be harvested from olfactory mucosal biopsies and proliferated to some extent, but the number of cells is limiting and mucosal-derived OEGs are not good a promoting axon regeneration [65]. An operation to obtain bulbar OEGs through an orbital route was developed, and a trial of this method in a patient was accompanied by sufficient sensory and motor recovery that further experimental treatments are planned [127].

Various other permissive cell types have been grafted into the spinal cord, including embryonic tissue, glial cells and stem cell types enabling some regeneration and functional recovery. Bone marrow stem cells have been widely used to protect and stimulate regeneration. Implanted into the spinal cord or introduced via blood vessels these cells have been neuroprotective, have improved functional recovery and in many cases stimulated regeneration of spinal axons [7, 33, 110, 117, 136]. The promising animal results led to a clinical study in which autologous cells were infused via a local spinal artery: the study demonstrated safety and good recovery in some patients, but no overall statistically significant improvement [126].

A remarkable advance has been the stimulation of regeneration of host axons by implants of embryonic spinal cord or spinal cord progenitors. Embryonic CNS tissue of the right stage is clearly permissive to axon growth, because it supports axonal ingrowth and formation of connections as part of normal development. Embryonic spinal cord transplants into the adult spinal cord attracted ingrowth of spinal cord axons from various neuronal types, and occasionally growth through the graft and out the other side back into the host cord [17, 108]. Axon ingrowth was greater in newborn animals, and there was also some specificity in that axons would not generally grow into non-target embryonic tissue such as hippocampus [16, 17]. The idea of foetal transplantation has recently been revived with improved methods and embryonic progenitor cells rather than whole transplants. The host-graft interface and the survival of cells has been improved by embedding the transplant in a fibrin/collagen gel containing multiple growth factors, and improved methods for progenitor production and characterisation and tracing of connections has enabled new levels of analysis. When the grafts are of spinal cord origin and the correct embryonic developmental state, prolific ingrowth of neurites is seen [85]. Of particular interest is the extensive ingrowth of fibres from the corticospinal tract, which is usually refractory to regeneration but able to sprout extensively after axotomy. It is not clear if the innervation of the grafts is very extensive sprouting or long-distance regeneration, but the processes are able to make many synaptic connections. Particularly exciting is the finding that the ingrowing axons show great specificity in the connections that they make with graft neurons. The injured motor and sensory axons reconnect with appropriate graft neurons, choosing neuronal types that normally receive either sensory or motor inputs [37], and even within the connections of corticospinal axons, the processes connect very precisely to the types of spinal interneuron that would normally receive corticospinal inputs [73]. Over time the graft neurons mature to express markers associated with adult neurons. It is not clear what the guidance mechanisms here would be, but clearly regenerating adult axons are able to select very precisely the correct type of neuronal target. These same neuronal types are able to grow axons that exit the graft and can grow for considerable distances down the host spinal cord, making connections with host neurons (see later). These embryonic grafts are therefore able to function as relays, receiving regenerated host axons as inputs and sending connections back to the host distal to the injury as projections. This relay behaviour is presumably the foundation of the ability of the grafts to restore function to the injured spinal cord.

The concept of the inhibitory CNS post-injury environment dominated research into CNS regeneration for many years. This work has been reviewed many times, so a brief account appears below. A number of inhibitory molecules are present in the CNS environment, some of them upregulated or released after injury. The main inhibitors are NogoA which is present on oligodendrocytes, chondroitin sulphate proteoglycans (CSPGs) are produced mainly by glia, myelin-associated glycoprotein (MAG) is produced by myelinating glia, Oligodendrocyte myelin glycoprotein (OMgp) by oligodendrocytes, semaphorin 3A mainly by perivascular and meningeal cells and tenascin-C from astrocytes [44]. The majority of research in this area has focused on NogoA and its receptor and CSPGs. Blocking the effects of NogoA with an antibody enabled regeneration and functional recovery in rodent and monkey models of spinal injury [118] and a phase 1 clinical trial has been completed with phase 2 planned to start soon. The Nogo receptor has been a focus, which is acted upon by NogoA, MAG and OMgp and signals via RhoA. Many animal studies of receptor knockouts and blockers have shown regeneration and functional recovery, and a receptor decoy is planned to enter early stage human trials [141]. CSPGs are upregulated around CNS injuries, produced mainly by astrocytes and oligodendrocyte precursors. There are several CSPG core proteins, all bearing serine-linked sulphated glycosaminoglycan (CS-GAG) chains. It is the CS-GAG chains that are the main source of inhibition of axon regeneration, so most studies have focused on digesting these with the bacterial enzyme chondroitinase ABC. CS-GAG digestion, inhibition of synthesis or blockade of the PTPσ CS-GAG receptor have all led to some axon regeneration and also functional recovery [23, 24, 134], although the functional recovery may be mainly due to the reactivation of plasticity that comes with removing CSPGs from perineuronal nets around GABAergic inhibitory neurons [123]. A trial of chondroitinase ABC treatment in canine patients with spinal cord injury demonstrated a useful restoration of walking and standing function [62]. The overall observation from neutralizing or removing inhibitory molecules or their receptors is that some axon regeneration and sprouting can be enabled together with reactivation of plasticity, but the axon regeneration is a long way short of complete repair. Some axons may regenerate sufficiently to cross a small lesion, and having done so they may connect to interneurons allowing their influence to be relayed down the spinal cord. None of the inhibitory molecules are absolute blockers of regeneration, and an excess of permissive molecules or axons with high growth potential will enable axon regeneration in their presence [4]. Treatments targeted at inhibitory molecules can certainly form part of a treatment for spinal cord injury, with their effect on promoting plasticity being particularly helpful in functionally incomplete lesions. By themselves these treatments can be expected to promote partial recovery of function after CNS damage, but they will not be sufficient for complete recovery.

## Intrinisic Control of Regeneration

In the years following the experiments from the Aguayo laboratory that demonstrated that the CNS environment is inhibitory for axon regeneration, the focus of research was on inhibitory molecules. Axons were assumed to be enabled by a permissive environment or blocked by an inhibitory one. Thus the reason why axons would regenerate in peripheral nerves and not the spinal cord was thought to be due to the environment rather than the properties of the axons. It became apparent that this was a much too simplistic view, and that different types of axon have very different abilities to grow and regenerate. The first evidence for this came from the study of embryonic CNS tissue grafted into the adult CNS. If the embryonic neurons were grafted at around the stage of maturity when they would normally be growing axons within the embryo, then axons from these grafts could grow for long distances through the supposedly inhibitory adult CNS, overcoming inhibition from the many inhibitory molecules. Thus grafts of spinal cord, striatum, substantia nigra, hippocampus could all send out axons into the adult CNS, and in many cases there was evidence that these axons could synapse with host neurons [108, 124, 146]. If the grafts came from human embryos, where axons continue to grow over a longer period than in the rodent CNS, then the axonal growth was particularly profuse [143]. Thus it appeared that axons growing from embryonic neurons were not subject to the inhibitory influences that could prevent regeneration of cut adult axons. A second example came from studying regeneration of the central and peripheral branches of sensory axons. The peripheral branch regenerates vigorously, restoring function in experimental animals and human patients. The central branch passes through the dorsal root, which is peripheral nerve territory containing Schwann cells and should therefore be permissive. Yet after dorsal root crushes the central branch shows a much less vigorous regenerative response that the peripheral branch [32]. Even within the adult CNS it became clear that some pathways such as the nigrostriatal tract have a relatively high ability to regenerate while others such as the corticospinal tract are very poor regenerators [22]. Studies of regeneration in the PNS revealed that peripheral axotomy initiates a programme of changes of gene expression with upregulation of a set of molecules termed RAGS (regeneration-associated molecules), while cutting CNS axons led to little or no upregulation of RAGS in their neurons [66, 91]. It was also possible to stimulate regeneration of CNS axons to some degree by treatment with an appropriate neurotrophin to stimulate the axon and neuron [51, 130]. Together, these and other observations started to shift the balance of activity of CNS regeneration research towards the intrinsic regenerative properties of neurons and their axons: current research in this area is described below.

## Neurons Lose Regenerative Ability as They Mature

As described above, embryonic neurons transplanted into the adult CNS will usually grow axons within the adult host CNS. When the grafts are taken from embryonic basal forebrain or striatum the neurons are typically at the stage at which they are growing axons in the embryo, and their processes are mostly cut or stripped off during the preparation, so the process growth is regeneration [18, 122]. However transplants of embryonic spinal cord progenitors from E14 rats or spinal cord-directed neural precursors differentiated from stem cells, transplanted before final differentiation and process outgrowth, are also able to grow prolific axonal projections that travel most of the length of a rat spinal cord or, in the case of human spinal precursors grafted into non-human primates, for up to 50 mm [85, 98, 113]. Thus embryonic neurons committed to differentiate into cells appropriate for their graft site have a remarkable ability to grow their axons into adult host CNS. This growth occurs in both white and grey matter, and appears to be little influenced by the inhibitory molecules that block regeneration by mature adult axons. There is a clear developmental window during which grafted cells will grow axons into adult host CNS, which ends as the neurons start to make connections in the embryo: intrinsic regenerative ability is lost with neuronal maturation. The axons make synaptic connections with many types of host spinal neuron, including motoneurons [86]. Over time the neurons mature and express adult neuronal markers [84]. The ability of grafted spinal cord and other CNS neurons to grow neurites into the adult host CNS is restricted to embryonic neurons at the appropriate developmental stage (generally before they have formed synapses). However sensory neurons, which retain the ability to regenerate their axons into adulthood, also retain the ability to grow axons when grafted into the adult spinal cord [35].

Changes in axon growth with maturity can be reproduced in tissue culture models. Retinal ganglion cells lose their ability to grow axons with maturity as a result of signals from the retina and retinal amacrine cells [50]. Cortical neurons cultured from E18 rats are at the developmental stage when growth of projection axons into the spinal cord and other brain regions is starting. When these neurons are placed into culture they quickly grow dendrites and an axon, and if the axon is then cut with a laser it regenerates in around 80% of cases. Over the ensuing weeks the neurons grow long processes, develop many synapses, become spontaneously electrically active and undergo gene expression changes to an adult pattern. When the axons of these mature neurons are cut the percentage that regenerate becomes progressively smaller with maturity [71]. The axons of mature DRG neurons continue to regenerate when cut, as in vivo, but the mode of growth changes from long straight axons to multiple branches [129].

Why do neurons lose axon regeneration as they mature? Research has focused on two areas, the axons themselves and the genetic and epigenetic changes that drive changes in the molecules present in the neurons. The two approaches are obviously linked, and axonal changes must be driven by changes in gene expression, but the research questions are easier to discuss separately.

## Axonal Mechanisms

### The Axonal Surface

**Fig. 1:**
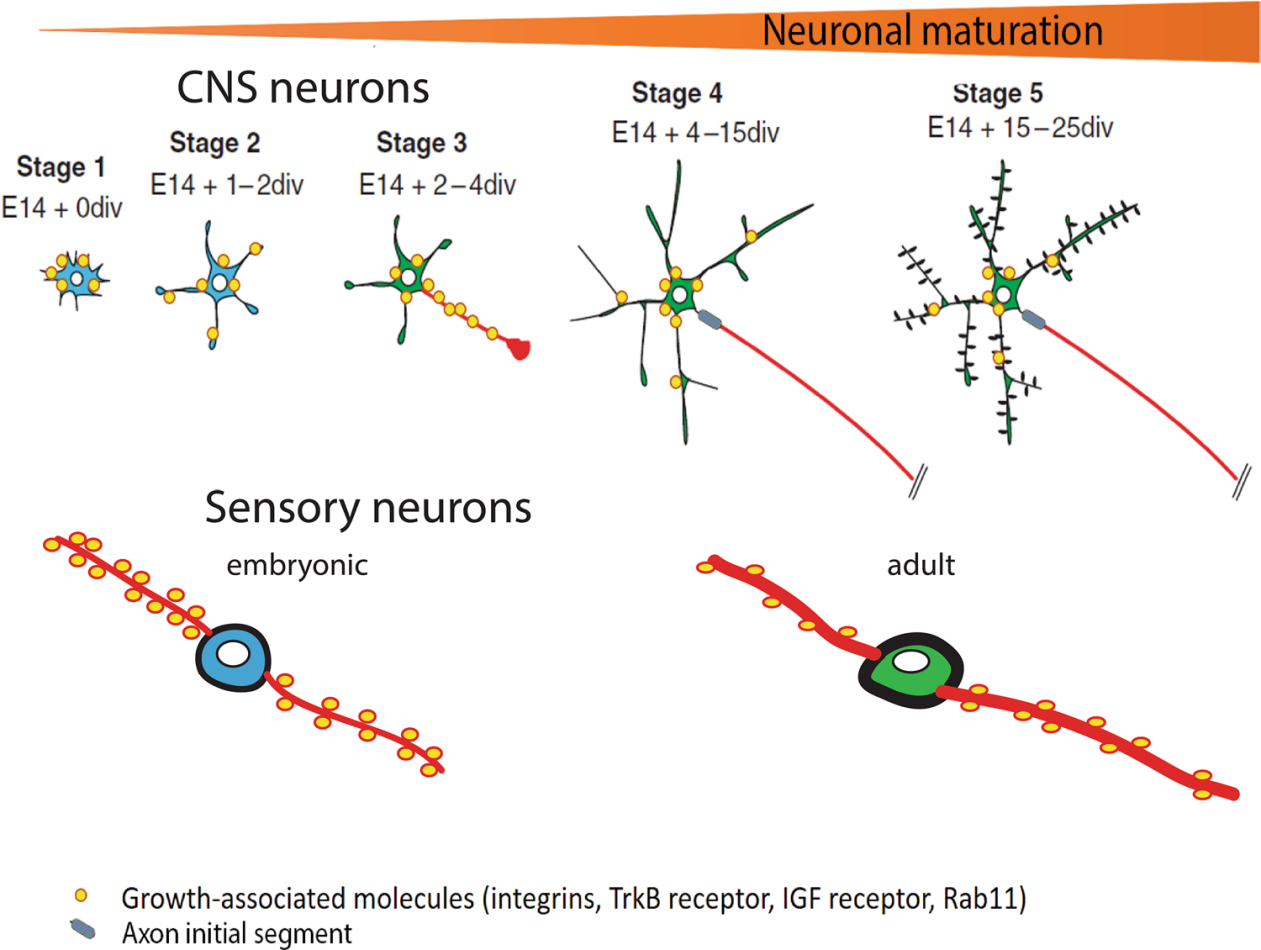
Changes in axonal transport of growth-related molecules such as integrins and the Rab11 vesicles that transport them. During developmental growth all neurons transport growth-related molecules into both axons and dendrites. As CNS neurons mature they become partitioned into cell body, dendritic and axonal domains. This partitioning achieves specialization of axons through selective transport of molecules, and many growth-related molecules are now excluded from axons. Sensory neurons are not partitioned to the same degree and growth-related molecules continue to be transported down axons in adulthood

Axons communicate with their environment through cell surface adhesion, receptor, channel and mechanosensitive molecules, and any axon growth must involve a dialogue with the environment via the surface. Key molecules are cell surface adhesion molecules which enable growing axons to exert traction on their environment and signal across the membrane, and growth factor receptors which drive many of the key axon signalling pathways. Regenerating axons penetrate the extracellular matrix, so integrins which bind to extracellular matrix glycoproteins are essential for regeneration. In the PNS several integrins are upregulated during regeneration and are necessary for axon growth [104]. In the CNS overall integrin expression falls with maturity, and integrins become selectively excluded from axons (Fig. 1), leading to these molecules being at very low levels or absent on CNS axons. The main glycoproteins in the mature CNS extracellular matrix are tenascin-C and osteopontin, both of which are upregulated after injury. The migration-promoting integrin that recognizes these ligands is α9β1 which is expressed during development but downregulated in both CNS and PNS neurons in adulthood [5]. In addition, the two CNS inhibitory molecules NogoA and CSPGs both inactivate integrins [61, 128], so the appropriate activated integrins are not available to enable axon regeneration. Viral expression of α9 integrin together with the integrin activator kindlin-1 in DRG neurons enabled long distance regeneration of sensory axons in the adult rat spinal cord. This demonstrates the principle that providing axons with an appropriate adhesion molecule for their environment can enable them to grow. However this strategy will not work without modification for long-tract CNS neurons, because the integrins are selectively excluded from their axons (except for some optic nerve axons) [6]. Knowledge of the presence of other adhesion molecules on mature CNS axons is limited. Type 1 and 2 cadherins and neurexins are present in synapses, so they may well be present on axon shafts, and other adhesion molecules such as L1 and NCAM are present during development, but L1 protein is not detectable in the corticospinal tract of adult mice, even when overexpressed [67]. Growth factor receptors are drivers of axon signalling which can promote growth, but here again some key receptors are excluded from corticospinal axons. Immunostaining for both IGF receptor and TrkB showed them to be present in proximal corticofugal axons near the cell body, but absent in the rest of the axon [58, 59]. Yet these receptors are present on developing axons and on the regeneration-competent axons of peripheral nerves. We can conclude, therefore, that mature CNS axons are lacking some of the key receptors that can enable regeneration (Fig. 1). Many adhesion and receptor molecules are present on the cell surface in the context of lipid-rich microdomains (rafts), which bring them together with signalling and other molecules that act in cis as co-factors. Reggies/flotillins are raft organizers which are strongly upregulated in regenerating fish and frog optic nerves, but not after optic nerve damage in mammals. Overexpression of reggie-1/flotillin-2 considerably increased regeneration in the optic nerve. Another related mechanism relates to gangliosides, glycosphingolipids which are raft components and organizers. In peripheral nerve axons, the membrane sialidase Neu7 is activated by axotomy to convert much of the ganglioside to pro-regenerative GM1, but this does not occur in adult retinal axons. Treatment with sialidase can restore regeneration to the ganglion cell axons in vitro and to spinal cord axons in vivo [70, 145]. Overall, it is clear that the surface of mature CNS axons lacks many of the growth-related receptors and structures that are present on the same axonal types during development, and on regeneration-competent axons in peripheral nerves.

### Axonal Transport

In the previous section the selective exclusion of adhesion molecules and growth factor receptors from CNS axons is described. How does this happen? Selective transport of molecules within neurons is part of their differentiation and maturation, and many types of molecules are involved [53]. As neurons become specialized for their purpose, dendrites and axons take on different functions, anatomy and composition. In order to give axons and dendrites these differences, different molecules have to be sent there. A complex set of mechanisms has therefore developed to send molecules and transport vesicles to the correct place. Some molecules are directed into axons or dendrites directly from the Golgi, but many are inserted into the membrane of the cell body first, then internalized and distributed through the recycling pathway (transcytosis) [75]. A recent genome-wide screen for regeneration-related molecules has identified transport-related molecules and particularly Rab GTPases (molecules that mark and target transport endosomes) as determinants of axon regeneration [119]. The differentially distributed molecules that have mainly been studied are integrins, trks, transferrin receptors (excluded from mature axons), L1 (transported into immature axons), glutamate receptors (transported to dendrites). Of these, integrins are particularly associated with axon growth so I will focus on them here. Integrins are present in PNS axons throughout life where they participate in regeneration. In the developing CNS integrins are transported into growing axons and are involved in axon growth. In the adult CNS integrins are excluded from the axons of cortical and red nucleus projection axons (Fig. 1), but a few optic nerve axons contain integrins. Even when the integrins are overexpressed they are still excluded from axons [6]. In cultured CNS neurons, cortical and substantia nigra neurons integrins are present in immature axons, but become excluded as neurons mature [47], and some integrins are present on retinal ganglion cell axons cultured from adult retina [137]. Endogenously-expressed integrins are present at low level, and they do not progress past the axon initial segment in cultured cortical neurons, but if the integrins are expressed at a high level there is some diffusion into the proximal axons, but the integrins are actively transported out of the axons back to the cell body. The integrins are mainly transported around neurons in recycling endosomes marked by the GTPases Rab11 and Arf6, and Rab11 vesicles are also excluded from the axons of mature neurons [71]. Live imaging of either integrins or Rab11 shows a change in transport with maturity. In immature neurons the relatively large amount of integrin and endosomes spend roughly equal amounts of time moving anterogradely and retrogradely, and this is seen also in DRG axons. As the axons mature mostly retrograde transport is seen, presumably removing any integrin that has diffused into the proximal axons [40, 47, 71]. The Rab11 and Arf6 endosomes are part of a complex which also contains the transport adapters JIP3/4. The activation state of Arf6 determines whether the complex will attach to dynein (for retrograde transport) or kinesin (for anterograde transport) [93]. The developmental event that changes the directionality of transport appears to be the upregulation of the Arf6 GEF (activator) Efa6, which accumulates at the axon initial segment and leads to Arf6 throughout the axon being in the activated (retrograde) state. Removal of Arf6 or expression of an Arf6 GAP (inactivator) allows integrins back into the axons and restores their ability to regenerate in the in vitro model; in vivo testing is in progress. In PNS axons, although Efa6 is present there is also a large amount of the Arf6 GAP ACAP1 which maintains anterograde transport [41]. Overall, the exclusion of adhesion molecules and growth factor receptors from mature axons is a major cause of their loss of regenerative ability. Restoring integrins to axons restores regeneration, but selective transport of other receptors needs to be examined in more detail to find out how to restore them to axons. Both IGFR-1 and TrkA and TrkB are found in Rab11 endosomes [10, 63, 76, 111], so some of the same rules that determine integrin localization may apply. Integrins, IGF, BDNF and NGF are all powerful enablers of axon regeneration, so ensuring sufficient levels in CNS axons would be a powerful stimulus to regeneration.

### Axonal Signalling

Growth is controlled by various signalling pathways, particularly those downstream of growth factor receptors (ERK, AKT), integrins (FAK), and the small GTPases and calcium. Signalling pathways affect all the mechanisms that control growth, including the polymerization and motor activity of the cytoskeleton, transport, trafficking, membrane addition, gene expression and epigenetics. There have been a large number of studies of signalling in axon regeneration using the various regeneration models in rodents, *C. elegans*, *Drosophila* and various in vitro models. A few signalling mechanisms have proven to be involved in regeneration across all these models. RhoA came to prominence because it transpired that several inhibitory pathways signalled via this route, particularly NogoA and CSPGs, so blocking RhoA should neutralize the effects of these regeneration blockers. Some bacteria produce an enzyme C3 transferase which inactivates Rho by attaching an ADP-ribose group. A cell permeable form of this enzyme was effective in promoting axon regeneration in both the optic nerve and spinal cord [77], and Rho kinase inhibitors were also effective [2, 26]. Growth factor receptors activate PI3 kinase which phosphorylates PIP2 to generate PIP3 which has a powerful stimulatory effect on axon growth and other forms of cell migration. PIP3 has a large number of effects, acting on the many proteins with PH or FYVE domains. Many of the effects are through activation of the AKT-mTOR pathway, but there are also various effects on transport and trafficking [56]. The level of PIP3 needs to be closely controlled because of its many and powerful effects, and this is achieved by balancing synthesis by PI3Kinases and dephosphorylation by PTEN and SHIP [72]. Deleting PTEN is therefore a powerful way of increasing and prolonging PIP3 signalling in the places where it is generated. PTEN knockout or knockdown has a strong effect on promoting axon regeneration in the optic nerve, and also in the spinal cord [82, 100]. However, the effect of PTEN knockout on regeneration is much more marked in young animals than in adults, which may reflect the maturity-related decline in receptor transport into CNS axons, meaning that there are fewer receptors to activate PI3kinase and generate PIP3. Importantly, PTEN knockdown has had synergistic effects with a variety of other regeneration-promoting treatments. PTEN knockout has a powerful oncogenic effect, so any practical use of this method will have to be carefully targeted to neurons. The kinase DLK was identified in *C. elegans* as a required component for axon regeneration [55, 144]. In the absence of the enzyme axons usually fail completely to regenerate. DLK regulates local protein synthesis in axons, particularly of the transcription factor CEBP-1 which is then retrogradely transported to affect gene expression, and it also influences microtubule dynamics, possibly via kinesin-13 [48, 144]. In mammals, DLK overactivity leads to axon overgrowth suggestive of microtubule overgrowth, and in the retina its activity is linked to both apoptosis and regeneration [142]. Calcium is central to responses to axotomy. After axotomy there is high calcium in the damaged axon tip, due to calcium leaking through the damaged membrane. This and other events lead to a more widespread release of calcium from internal stores, and the most cut axons fire rapidly for a long period allowing yet more calcium to enter the whole axon and cell body [21]. These calcium events initiate metalloproteinase activity leading to formation of the retraction bulb, activate various signalling pathways, and also initiate the local translation of proteins which is central to retrograde signalling [109]. If sensory axons are cut in a calcium-free medium they mostly fail to regenerate [139]. Recently the voltage gated calcium channel Alpha2delta2 has been identified as a repressor of axon regeneration in sensory neurons, while deletion of the gene promotes regeneration. Importantly pregabalin, a widely used pain medication, blocks the channel and promotes sensory axon regeneration in the spinal cord and peripheral nerves [129]. In addition to the examples above, deletion or inhibition of many of the major signalling pathways reduces axon regeneration.

Neurotrophins and other trophic factors are powerful activators of axonal signalling as long as their receptors are present. NT3 has had beneficial effects on corticospinal regeneration in the spinal cord [20] and also on regrowth of proprioceptive axons [105], and NGF has a strong effect on promoting sprouting of sensory axons in the spinal cord, but this is not practically useful because it can lead to chronic pain [112]. IGF-1 together with ostopontin was a strong promoter of optic nerve regeneration [83]. Regeneration in the optic nerve has also been increased by CNTF, IL-6, and other trophic factors [78, 81]. Stimulation of axon regeneration by expression of activated integrin, described earlier, depends on signalling via FAK [28]. An alternative to activating receptors is to intervene directly in the signalling pathway. Overall, any treatment that promotes axon regeneration will modulate signalling pathways in axons. In order to drive regeneration with receptor-ligand-induced signalling, it will first be necessary to get the receptors to where they are needed at the ends of axons. Direct signalling interventions have effects on all types of cell, so treatments of this type will have to be carefully targeted and controlled.

### Local Protein Synthesis

Axon regeneration can only occur if the materials for building axons are provided. This presents a problem because some axons are very long and axonal transport is slow- around 25 cm per day at its fastest, so for signals to reach the cell body, initiate protein synthesis and for those new proteins to reach the cut axon tip can take a considerable time. Regeneration would be more efficient if proteins could be synthesized in the axons, and for some axons this certainly occurs. This applies also to signals from the damaged axon to the cell body, which rely on synthesis of proteins at the injury site [109]. In peripheral axons mRNAs are selectively transported in axons and the machinery for local synthesis and control is present. If local protein synthesis or local protein degradation is inhibited then axon regeneration is much less efficient [139]. The mechanisms behind local synthesis are complex. A restricted set of mRNAs are transported into sensory axons [54], and they are transported in association with a variety of RNA binding proteins recognized by motifs on the RNAs, and linked to kinesin [116]. The RNAs can be protected from translation as they are transported, for instance through binding to ZBP-1 through linkage to the cytoskeleton [13, 116] and through the stress granule protein G3BP1 [115]. Importantly, the ribonucleoproteins can be localized in the growth cone by interactions with receptors for attractant and repellent stimuli [79, 102]. This allows local translation to participate in growth cone guidance and responses to the environment. Local translation can also drive growth cone signalling through local translation of mTOR (which also controls synthesis). Also produced are retrograde signalling molecules including importin beta and STAT3, which control the genetic response to axotomy (see below). The mRNAs that are translated include actin, tubulin, GAP-43 and the retrograde signalling molecules [36, 135]. Local protein synthesis is also a fundamental process behind the growth and guidance of axons in development [121]. An unresolved question is the extent to which local translation occurs in the long-tract axons in the spinal cord and elsewhere that are refractory to regeneration. In the CNS some axons that have the machinery for local translation can regenerate into Schwann cell-containing grafts [68], but many of these are probably the central branches of sensory axons. Local translation occurs in adult mammalian retinal ganglion cell axons (which have relatively high regenerative capacity) [121]. If local translation is absent or marginal in damaged CNS axons, it is probably the transport and trafficking of the mRNA-protein complexes and the translational machinery such as ribosomes that will need attention.

### The Axonal Cytoskeleton

Axon growth is driven by the cytoskeleton. Actin dynamics in the filopodia and lamellipodia of the growth cone, linked to the environment via adhesion molecules, exerting traction on the axon via myosin and reinforced by microtubules are the motor of growth. Many of the processes discussed above are happening in order to control cytoskeletal events. Transport and trafficking also occur in association with actin and tubulin [87]. Our knowledge of the detailed cytoskeletal events that follow axotomy comes mainly from observing the very large axons of *aplysia*, regeneration in *c. elegans* axons and axotomy of mammalian axons in vitro and in vivo [21]. The first event is withdrawal of the cut end of the axon followed by formation of a retraction bulb. These events are precipitated by a large increase in calcium, entering the damaged axon from the outside and released from internal stores [69]. The calcium triggers various proteases which digest cytoskeletal elements and the submembranous layer of actin and spectrin. Inside the retraction bulb are randomly arranged microtubules and accumulations of transport vesicles [30, 38, 39]. Soon new microtubules start to be formed, visualised by an increase in the number of tubules capped by end-binding proteins, and actin filaments reappear [30]. In axons that are capable of regeneration a new growth cone is formed that is similar to developmental-type growth cones, but in regeneration-incompetent axons (which mainly means those of the mammalian CNS), the retraction bulb continues to be dynamic for many hours, but does not generate a new growth cone [71, 132, 139]. For regeneration to proceed, new cytoskeletal molecules are required. In some axons these can be produced by local translation (see above) [36, 140], for polymers such as actin filaments and microtubules local recycling can occur, and in axons that regenerate there is an overall increase in axonal transport bringing many molecules to the growing axon tip [8, 88, 97]. Modification of the cytoskeletal events following axonal damage can be beneficial. Taxol and epothilone B are microtubule-stabilising compounds which can reduce retraction and retraction bulb formation, and can enhance regeneration of sensory and serotinergic CNS axons after spinal cord injury. Conversely depolymerising microtubules with nocodazole turns cut sensory axon tips into sterile retraction bulbs that fail to regenerate [39, 114]. There are many controls of cytoskeletal behaviour, but some molecules stand out. Efa6 was mentioned above because of the part it plays in excluding molecules from axons. In *C.elegans* it has a direct effect on restraining microtubule dynamics. A large screen to find molecules that affect regeneration [29, 96] came up as expected with many molecules that reduce regeneration if they are absent, but much rarer are molecules such as Efa6 whose absence enhances regeneration and therefore are functional and regeneration suppressors. Efa6 clearly gives excessive microtubule stabilisation. A number of molecules affect microtubule dynamics by binding to them and having the ability to sever the microtubules, spastin atlastin fidgetin and katanin are examples. The level of these molecules is critical: in *Drosophila* if levels of spastin and atlastin are too high or too low regeneration fails [107], and fidgetin is a regeneration inhibitor [89]. Kinesins are generally thought of as transport molecules, but some can also affect microtubule dynamics. Kinesin-13 is a tubulin depolymerizing molecule regulated by DLK-1 (see above), which can restrict microtubule growth after axotomy, and kinesin Kif3C is binds to microtubule end-binding proteins where it affects microtubule dynamics and is necessary for regeneration in mammalian sensory axons [52]. Microtubules are modified in various ways, particularly by tyrosination and acetylation. Tyrosinated microtubules turn over faster than detryrosinated, and acetylation is associated with stable older microtubules and deacetylation increases microtubule dynamics. In growing axons the more dynamic microtubules are seen at the growth cone. The histone deacetylase HDAC5 is an important player in peripheral nerve regeneration. After axotomy it is localized to the cut axon by association with the actin-binding protein filamin A, and at the same time it leave the nucleus enabling epigenetic changes (see later). In the axons it decetylates microtubules, promoting microtubule dynamics and assisting regeneration, but a similar effect in CNS neurons has not been found [31]. Growing axon tips have tyrosinated dynamic microtubules [12, 39]. Reduced detyrosination has been associated with enhanced regeneration in the optic nerve, sciatic nerve and in sensory axons through the interaction with Kif3C [49, 52]. Overall, the cytoskeleton is the motor of axon growth, and there are several types of intervention that can affect regeneration. Of these the most practical is microtubule stabilization with epothilone-B, which has the added advantage that it modifies the glial reaction to injury.

## Genetics and Epigenetics

Damage to peripheral nerves results in the initiation of a regenerative programme, with upregulation of many genes. A peripheral nerve crush will initiate this genetic programme, and a subsequent lesion of sensory axons in the spinal cord will lead to a more vigorous regeneration response than the same spinal lesion without a previous peripheral lesion [95]. Similarly, a second lesion to the peripheral axons will lead to faster regeneration than if there had been no previous lesion. This is known as the conditioning effect. Damage to the central branch of the sensory axons alone does not initiate this same pattern of upregulation of gene expression, and neither do lesions to intrinsic CNS axons. The PNS axotomy response and the conditioning effect that it produces has therefore been used as a model for the genetic changes that should drive enhanced regeneration of axons [11].

The regeneration response is driven by retrograde signals from the site of axotomy, and can also be initiated to some extent by local inflammatory and cytokine effects in the dorsal root ganglia. The early retrograde signal is calcium. Calcium is initially greatly increased through leakage at the injury site, and this leads to more widespread release of calcium from internal stores. The lesion and its effects also usually lead to rapid firing of the damaged axons, leading to further calcium entry. These stimuli lead to expression changes in the cell body, and also activation of signalling pathways and initiation of local translation at the injury to produce retrograde signals, leading to retrograde transport of STAT3, DLK, JNK in association with importin-beta and vimentin [15]. Several cytokines, including IL-6, LIF and CNTF are also upregulated [25, 120, 125]. The result is upregulation of more than 1000 genes in sensory neurons. However axotomy can also have non-specific negative effects on translation through released ribonucleases, which can modify rough endoplasmic reticulum, degrade ribosomes and monoribosomes [94]. Of these interest has focused particularly on transcription factors that may orchestrate the programme. A recent bioinformatics study has identified a core group of these, ATF3, EGR1, FOS, JUN, MYC, RELA, SMAD1 and STAT3 that are expressed and positioned in the control network to be the controllers of the axotomy response [27]. The large majority of these changes in gene expression do not occur after axotomy of the CNS branch of sensory axons or after lesions of intrinsic CNS axons. The logical next step is to express one or more of these transcription factors in sensory or CNS neurons in order to activate a regeneration programme and enable axon regeneration in peripheral nerves and in the CNS. Expression of single transcription factors has led to some increase in regeneration, so expression of multiple factors has been tried. For instance expression of ATF3 increased speed of regeneration in peripheral nerves, but marginally enabled regeneration in the spinal cord. A combination of four transcription factors, ATF3, SMAD1, STAT3 and c-Jun was no more effective that ATF3 alone [42]. In the CNS the results of transcription factor manipulation have shown some increase in regeneration. Of the transcription factors listed above involved in the sensory neuron axotomy response, overexpression of an active form of STAT3 enhanced corticospinal tract sprouting and slightly increased optic nerve regeneration [74, 90], activation of the SMAD1 pathway enhanced regeneration in the optic nerve and spinal cord [131]. The conclusion is that the injury response in sensory neurons clearly enhances regeneration, because it enables increased growth when sensory axons are cut in the spinal cord and faster regeneration in peripheral nerves. However, simply expressing some of the transcription factors does not have as strong an effect on regeneration as peripheral nerve lesions, and expressing the transcription factors in CNS neurons has only a small effect on their ability to regenerate.

A similar logic applies to the study of genetic changes during neuronal maturation. It is clear that neurons lose intrinsic regenerative ability with maturity, so study of the genetic changes during maturation and attempting to reverse these changes by expressing or knocking down transcription factors whose expression has changed during maturation is a logical step. Research activity in this area has focused on the Kruppel-like factor (KLF) family of transcription factors. Several of these change their expression during maturation. Deletion KLF9 or overexpression of KLF6 or KLF7 has positive effects on regeneration in the spinal cord and optic nerve [9, 19, 133]. Also p53 expression increased corticospinal regeneration [45], but again these interventions do not restore the neurons to their embryonic level of regenerative ability.

The results above are sufficient to demonstrate that manipulating transcription factor expression in mature neurons can have effects on axon regeneration. However, the effects are relatively modest. Expression of only two to four transcription factors in fibroblasts is sufficient to return them to a stem cell state as iPS cells, but rejuvenating neurons seems to be much more difficult. An important difference is that the fibroblasts are dividing and neurons are not. During mitosis the chromatin is opened up and histones removed, making many areas of chromatin accessible that were previously hidden. This has been called mitotic advantage. When non-dividing nuclei are placed in oocytes their pattern of gene expression changes much less than dividing nuclei, and many transcription factor binding sites only become accessible during mitosis. A major mechanism for masking transcription factor binding sites is binding to the chromatin of modified histones [92], and methylation of DNA is also involved. In mature neurons a similar masking of chromatin sites appears to prevent expressed transcription factors from having the intended effects on regeneration. Recent research has started to address these issues. There is evidence that the expression of the regeneration-associated genetic programme in sensory neurons, triggered by axotomy, is made possible by epigenetic changes that open up interaction sites. There are many histones, each subject to many types of modification that affect their chromatin binding. The best understood regulation of histones is by acetylation, mediated by histone acetyltransferases (HATs) and histone deacetylases (HDACs). Two examples of changes in histone acetylation after peripheral axotomy are understood in some detail. HDAC5 is usually found in sensory neuron nuclei, but after axotomy it is exported into the cytoplasm and the axons. The trigger is calcium via protein kinase C. HDAC5 in the axon affects microtubule acetylation as described above, while removing HDAC5 from the nucleus enables expression of the early response genes of the regeneration transcription programme, and without HDAC5 export regeneration is inhibited [31]. There are also changes in HATs. The HAT histone acetyltransferase p300/CBP-associated factor (PCAF) is activated by ERK signalling after injury, and the PCAF then modifies histones at the promoters of some key regeneration-related genes. Without PCAF activity the conditioning effect and regeneration of sensory axons in the spinal cord is not seen [103]. Histone acetylation via Creb-binding protein (Cbp) is also the link between rehabilitation activity and expression of the regenerative programme in DRG neurons [64]. These changes in HDACs and HATs do not occur after axotomy in the CNS. A recent focus has been to try to rejuvenate mature CNS neurons, bringing back the regenerative ability that they had during embryogenesis, and, as described above, some success has been obtained by modulating the expression of KLF transcription factors and combining KLF9 deletion with JNK3 produces an additive effect [9]. However the general finding has been that it has not been possible to reawaken the axon growth programme through expression of transcription factors. The problem appears to be one of epigenetics. During CNS cortical neuronal maturation there is a major change in the accessible regions of the genome, with only 30% of accessible sites being available at both immature and mature stages. Many of the genes that become masked in adulthood were related to axon growth, while the genes that were available at both stages were mostly related to homeostasis, vesicular transport and ion transport. Two pro-regenerative transcription factors whose target genes are accessible in mature neurons in the cortex (KLF7 and STAT3) were successful at increasing corticspinal tract regeneration, while two factors with inaccessible targets (JUN and STAT3) did not promote regeneration when expressed [138]. What might drive these maturational changes in gene expression and and epigenetics? Many of the changes described above occur at the same time as axons have reached their targets and synapses are forming and maturing. Signalling to the nucleus from the postsynaptic side of synapses in dendrites or from the presynaptic side in axons is a strong candidate, and several signalling pathways that affect neuronal gene expression have been identified which would be candidates for driving the neuronal maturation process [57, 99]. Overall, it appears that for neurons to express a regeneration transcriptional programme the right transcription factors must be expressed and present in the nucleus. However the presence of the transcription factors is not enough, the promoters and enhancers that drive the regeneration programme must be accessible. In sensory neurons the effects of axotomy reveal some of the key promoters and enhancers, so they can enter a regeneration-competent state. In CNS neurons many of the key promoters and enhancers are hidden, and the signalling events that follow CNS axotomy do not initiate events to reveal them. In order to drive regeneration of CNS neurons we will need to understand how to manipulate these epigenetic events that block transcription of the key regeneration genes.

## Perspectives

I started this article by asking why it has been so difficult and taken so long to find out how to regenerate axonal pathways in the CNS. The sections above demonstrate how complex regeneration is, and how many interventions have had significant but insufficient effects on stimulating regeneration. Why has the problem turned out to be so complicated? When research on this subject started to investigate mechanisms in the 1980s there was an assumption that regeneration would largely recapitulate development, and that one or two inhibitory mechanisms would need to be corrected. It has turned out that regeneration is very different to embryonic development, and that there are a large number of mechanisms that block regeneration and repair. There must be significant evolutionary benefits in restriction regeneration, as argued above, so evolution has gone about turning off regeneration in the usual way. Human engineers when they want to solve a problem look for a single elegant and powerful solution. Evolution hardly ever works this way. Because it has to work with random events that must give selective advantage, it tends to end up solving problems by creating several parallel mechanisms, often individually not very efficient, which together add up to a robust and failure-proof solution. It seems likely that turning off regeneration and repair has gone this way, with the many mechanisms above as the consequence. How are we to repair the damaged CNS? It will almost certainly not be necessary to address all the potential inhibitory mechanisms. For instance it is encouraging that the fairly simple single intervention of expressing an appropriate integrin and integrin activator was able to promote long distance sensory regeneration. For CNS neurons the problem is more difficult because of the suppression of the regeneration transcriptional programme, and because the neurons are partitioned to exclude many growth related molecules from mature axons. From a practical perspective it seems sensible to work both on the axons, restoring transport and trafficking of the key molecules, and on the transcriptional programme in cell bodies. If we can arrange for expression of the right molecules and then get them to the right place, then axons will regenerate.

## Abbreviations

ACAP1: Arf-GAP with coiled-coil, ANK repeat and PH domain-containing protein 1
Arf6: ADP ribosylation factor 6
CS-GAG: Chondroitin sulphate glycosaminoglycan
CSPG: Chondroitin sulphate proteoglycan
DLK-1: Mitogen-activated protein kinase kinase kinase dlk-1
Efa6: Exchange factor for Arf6
FAK: Focal Adhesion Kinase
GAP: GTPase activating protein
GEF: Guanyl-nucleotide exchange factor
JIP: C-Jun-amino-terminal kinase-interacting protein
KLF: Kruppel-like factor
MAG: Myelin-associated glycoprotein
Neu7: Membrane ganglioside Sialidase 7
OEG: Olfatory ensheathing glia
OMgp: Oligodenrocyte myelin glycoprotein
PIP3: Phosphatidylinositol (3,4,5)-trisphosphate
Rab11: Ras-related protein Rab11
Trk: Tropomyosin-related kinase (neurotrophin receptor

## References

[1] Adcock KH, Brown DJ, Shearer MC, Shewan D, Schachner M, Smith GM, Geller HM, Fawcett JW (2003). Axon behaviour at Schwann cell astrocyte boundaries. Manipulation of axon signalling pathways and glia can enable axons to cross. Eur J Neurosci.

[2] Ahmed Z, Berry M, Logan A (2009). ROCK inhibition promotes adult retinal ganglion cell neurite outgrowth only in the presence of growth promoting factors. Mol Cell Neurosci.

[3] Anderson KD, Guest JD, Dietrich WD, Bartlett Bunge M, Curiel R, Dididze M, Green BA, Khan A, Pearse DD, Saraf-Lavi E, Widerstrom-Noga E, Wood P, Levi AD (2017). Safety of autologous human schwann cell transplantation in subacute thoracic spinal cord injury. J Neurotrauma..

[4] Anderson MA, Burda JE, Ren Y, Ao Y, O'Shea TM, Kawaguchi R, Coppola G, Khakh BS, Deming TJ, Sofroniew MV (2016). Astrocyte scar formation aids central nervous system axon regeneration. Nature.

[5] Andrews MR, Czvitkovich S, Dassie E, Vogelaar CF, Faissner A, Blits B, Gage FH, Ffrench-Constant C, Fawcett JW (2009). Alpha9 integrin promotes neurite outgrowth on tenascin-C and enhances sensory axon regeneration. J Neurosci.

[6] Andrews MR, Soleman S, Cheah M, Tumbarello DA, Mason MRJ, Moloney EB, Verhaagen J, Bensadoun A, Schneider B, Aebischer P, Fawcett JW (2016). Axonal localization of integrins in the CNS is neuronal type and age dependent. Eneuro.

[7] Ankeny DP, McTigue DM, Jakeman LB (2004). Bone marrow transplants provide tissue protection and directional guidance for axons after contusive spinal cord injury in rats. Exp Neurol..

[8] Antonian E, Perry GW, Grafstein B (1987). Fast axonally transported proteins in regenerating goldfish optic nerve: effect of abolishing electrophysiological activity with TTX. Brain Res.

[9] Apara A, Galvao J, Wang Y, Blackmore M, Trillo A, Iwao K, Brown DP, Fernandes KA, Huang A, Nguyen T, Ashouri M, Zhang X, Shaw PX, Kunzevitzky NJ, Moore DL, Libby RT, Goldberg JL (2017). KLF9 and JNK3 Interact to suppress axon regeneration in the adult CNS. J Neurosci.

[10] Ascano M, Richmond A, Borden P, Kuruvilla R (2009). Axonal targeting of Trk receptors via transcytosis regulates sensitivity to neurotrophin responses. J Neurosci.

[11] Attwell CL, van Zwieten M, Verhaagen J, Mason MRJ (2018). The dorsal column lesion model of spinal cord injury and its use in deciphering the neuron-intrinsic injury response. Dev Neurobiol.

[12] Baas PW, Slaughter T, Brown A, Black MM (1991). Microtubule dynamics in axons and dendrites. J Neurosci Res.

[13] Bassell GJ, Singer RH (2001). Neuronal RNA localization and the cytoskeleton. Results Probl Cell Differ.

[14] Belen D, Aciduman A, Er U (2009) History of peripheral nerve repair: may the procedure have been practiced in Hippocratic School? Surg Neurol 72:190–193; discussion 193–194 10.1016/j.surneu.2008.03.030 18482755

[15] Ben-Yaakov K, Dagan SY, Segal-Ruder Y, Shalem O, Vuppalanchi D, Willis DE, Yudin D, Rishal I, Rother F, Bader M, Blesch A, Pilpel Y, Twiss JL, Fainzilber M (2012). Axonal transcription factors signal retrogradely in lesioned peripheral nerve. EMBO J.

[16] Bernstein-Goral H, Bregman BS (1993). Spinal cord transplants support the regeneration of axotomized neurons after spinal cord lesions at birth: a quantitative double-labeling study. Exp Neurol.

[17] Bernstein GH, Diener PS, Bregman BS (1997). Regenerating and sprouting axons differ in their requirements for growth after injury. Exp Neurol.

[18] Bjorklund A (2005). Cell therapy for Parkinson's disease: problems and prospects. Novartis. Found Symp.

[19] Blackmore MG, Wang Z, Lerch JK, Motti D, Zhang YP, Shields CB, Lee JK, Goldberg JL, Lemmon VP, Bixby JL (2012). Kruppel-like Factor 7 engineered for transcriptional activation promotes axon regeneration in the adult corticospinal tract. Proc Natl Acad Sci USA.

[20] Blesch A, Tuszynski MH (2001). GDNF gene delivery to injured adult CNS motor neurons promotes axonal growth, expression of the trophic neuropeptide CGRP, and cellular protection. J Comp Neurol.

[21] Bradke F, Fawcett JW, Spira ME (2012). Assembly of a new growth cone after axotomy: the precursor to axon regeneration. Nat Rev Neurosci.

[22] Brecknell JE, Haque NSK, Du JS, Muir E, Fidler PS, Hlavin M-L, Fawcett JW, Dunnett SB (1996). Functional and anatomical reconstruction of the 6-OHDA lesioned nigrostriatal system of the adult rat. Neuroscience.

[23] Burnside ER, Bradbury EJ (2014). Manipulating the extracellular matrix and its role in brain and spinal cord plasticity and repair. Neuropathol Appl Neurobiol.

[24] Burnside ER, De Winter F, Didangelos A, James ND, Andreica EC, Layard-Horsfall H, Muir EM, Verhaagen J, Bradbury EJ (2018). Immune-evasive gene switch enables regulated delivery of chondroitinase after spinal cord injury. Brain.

[25] Cao Z, Gao Y, Bryson JB, Hou J, Chaudhry N, Siddiq M, Martinez J, Spencer T, Carmel J, Hart RB, Filbin MT (2006). The cytokine interleukin-6 is sufficient but not necessary to mimic the peripheral conditioning lesion effect on axonal growth. J Neurosci.

[26] Chan CC, Khodarahmi K, Liu J, Sutherland D, Oschipok LW, Steeves JD, Tetzlaff W (2005). Dose-dependent beneficial and detrimental effects of ROCK inhibitor Y27632 on axonal sprouting and functional recovery after rat spinal cord injury. Exp Neurol.

[27] Chandran V, Coppola G, Nawabi H, Omura T, Versano R, Huebner EA, Zhang A, Costigan M, Yekkirala A, Barrett L, Blesch A, Michaelevski I, Davis-Turak J, Gao F, Langfelder P, Horvath S, He Z, Benowitz L, Fainzilber M, Tuszynski M, Woolf CJ, Geschwind DH (2016). A Systems-level analysis of the peripheral nerve intrinsic axonal growth program. Neuron.

[28] Cheah M, Andrews MR, Chew DJ, Moloney EB, Verhaagen J, Fassler R, Fawcett JW (2016). Expression of an activated integrin promotes long-distance sensory axon regeneration in the spinal cord. J Neurosci.

[29] Chen L, Wang Z, Ghosh-Roy A, Hubert T, Yan D, O'Rourke S, Bowerman B, Wu Z, Jin Y, Chisholm AD (2011). Axon regeneration pathways identified by systematic genetic screening in *C. elegans*. Neuron.

[30] Chisholm AD (2013). Cytoskeletal dynamics in *Caenorhabditis elegans* axon regeneration. Annu Rev Cell Dev Biol.

[31] Cho Y, Sloutsky R, Naegle KM, Cavalli V (2013). Injury-induced HDAC5 nuclear export is essential for axon regeneration. Cell.

[32] Chong MS, Woolf CJ, Turmaine M, Emson PC, Anderson PN (1996). Intrinsic versus extrinsic factors in determining the regeneration of the central processes of rat dorsal root ganglion neurons: the influence of a periperal nerve graft. J Comp Neurol.

[33] Cizkova D, Rosocha J, Vanicky I, Jergova S, Cizek M (2006). Transplants of human mesenchymal stem cells improve functional recovery after spinal cord injury in the rat. Cell Mol Neurobiol.

[34] David S, Aguayo AJ (1981). Axonal elongation into peripheral nervous system bridges after central nervous system injury in adult rats. Science.

[35] Davies SJA, Fitch MT, Memberg SP, Hall AK, Raisman G, Silver J (1997). Regeneration of adult axons in white matter tracts of the central nervous system. Nature.

[36] Donnelly CJ, Park M, Spillane M, Yoo S, Pacheco A, Gomes C, Vuppalanchi D, McDonald M, Kim HH, Merianda TT, Gallo G, Twiss JL (2013). Axonally synthesized beta-actin and GAP-43 proteins support distinct modes of axonal growth. J Neurosci.

[37] Dulin JN, Adler AF, Kumamaru H, Poplawski GHD, Lee-Kubli C, Strobl H, Gibbs D, Kadoya K, Fawcett JW, Lu P, Tuszynski MH (2018). Injured adult motor and sensory axons regenerate into appropriate organotypic domains of neural progenitor grafts. Nat Commun.

[38] Erez H, Malkinson G, Prager-Khoutorsky M, De Zeeuw CI, Hoogenraad CC, Spira ME (2007). Formation of microtubule-based traps controls the sorting and concentration of vesicles to restricted sites of regenerating neurons after axotomy. J Cell Biol.

[39] Erturk A, Hellal F, Enes J, Bradke F (2007). Disorganized microtubules underlie the formation of retraction bulbs and the failure of axonal regeneration. J Neurosci.

[40] Eva R, Dassie E, Caswell PT, Dick G, Ffrench-Constant C, Norman JC, Fawcett JW (2010). Rab11 and its effector Rab coupling protein contribute to the trafficking of beta 1 integrins during axon growth in adult dorsal root ganglion neurons and PC12 cells. J Neurosci.

[41] Eva R, Koseki H, Kanamarlapudi V, Fawcett JW (2017). EFA6 regulates selective polarised transport and axon regeneration from the axon initial segment. J Cell Sci.

[42] Fagoe ND, Attwell CL, Kouwenhoven D, Verhaagen J, Mason MR (2015). Overexpression of ATF3 or the combination of ATF3, c-Jun, STAT3 and Smad1 promotes regeneration of the central axon branch of sensory neurons but without synergistic effects. Hum Mol Genet.

[43] Fawcett JW (2018). The paper that restarted modern central nervous system axon regeneration research. Trends Neurosci.

[44] Fawcett James W., Schwab Martin E., Montani Laura, Brazda Nicole, MÜller Hans Werner (2012). Defeating inhibition of regeneration by scar and myelin components. Handbook of Clinical Neurology.

[45] Floriddia EM, Rathore KI, Tedeschi A, Quadrato G, Wuttke A, Lueckmann JM, Kigerl KA, Popovich PG, Di Giovanni S (2012). p53 Regulates the neuronal intrinsic and extrinsic responses affecting the recovery of motor function following spinal cord injury. J Neurosci.

[46] Fouad K, Schnell L, Bunge MB, Schwab ME, Liebscher T, Pearse DD (2005). Combining Schwann cell bridges and olfactory-ensheathing glia grafts with chondroitinase promotes locomotor recovery after complete transection of the spinal cord. J Neurosci.

[47] Franssen EH, Zhao RR, Koseki H, Kanamarlapudi V, Hoogenraad CC, Eva R, Fawcett JW (2015). Exclusion of integrins from CNS axons is regulated by Arf6 activation and the AIS. J Neurosci.

[48] Ghosh-Roy A, Goncharov A, Jin Y, Chisholm AD (2012). Kinesin-13 and tubulin posttranslational modifications regulate microtubule growth in axon regeneration. Dev Cell.

[49] Gobrecht P, Andreadaki A, Diekmann H, Heskamp A, Leibinger M, Fischer D (2016). Promotion of functional nerve regeneration by inhibition of microtubule detyrosination. J Neurosci.

[50] Goldberg JL, Klassen MP, Hua Y, Barres BA (2002). Amacrine-signaled loss of intrinsic axon growth ability by retinal ganglion cells. Science.

[51] Grill R, Murai K, Blesch A, Gage FH, Tuszynski MH (1997). Cellular delivery of neurotrophin-3 promotes corticospinal axonal growth and partial functional recovery after spinal cord injury. J Neurosci.

[52] Gumy LF, Chew DJ, Tortosa E, Katrukha EA, Kapitein LC, Tolkovsky AM, Hoogenraad CC, Fawcett JW (2013). The kinesin-2 family member KIF3C regulates microtubule dynamics and is required for axon growth and regeneration. J Neurosci.

[53] Gumy LF, Hoogenraad CC (2018). Local mechanisms regulating selective cargo entry and long-range trafficking in axons. Curr Opin Neurobiol.

[54] Gumy LF, Yeo GS, Loraine Tung YC, Zivraj KH, Willis D, Coppola G, Lam BY, Twiss JL, Holt CE, Fawcett JW (2010). Transcriptome analysis of embryonic and adult sensory axons reveals changes in mRNA repertoire localization. RNA.

[55] Hammarlund M, Nix P, Hauth L, Jorgensen EM, Bastiani M (2009). Axon regeneration requires a conserved MAP kinase pathway. Science.

[56] Hawkins PT, Anderson KE, Davidson K, Stephens LR (2006). Signalling through Class I PI3Ks in mammalian cells. Biochem Soc Trans..

[57] Herbst WA, Martin KC (2017). Regulated transport of signaling proteins from synapse to nucleus. Curr Opin Neurobiol.

[58] Hollis ER, Jamshidi P, Low K, Blesch A, Tuszynski MH (2009). Induction of corticospinal regeneration by lentiviral trkB-induced Erk activation. Proc Natl Acad Sci USA.

[59] Hollis ER, Lu P, Blesch A, Tuszynski MH (2009). IGF-I gene delivery promotes corticospinal neuronal survival but not regeneration after adult CNS injury. Exp Neurol.

[60] Houle JD, Tom VJ, Mayes D, Wagoner G, Phillips N, Silver J (2006). Combining an autologous peripheral nervous system "bridge" and matrix modification by chondroitinase allows robust, functional regeneration beyond a hemisection lesion of the adult rat spinal cord. J Neurosci.

[61] Hu F, Strittmatter SM (2008). The N-terminal domain of Nogo-A inhibits cell adhesion and axonal outgrowth by an integrin-specific mechanism. J Neurosci.

[62] Hu HZ, Granger N, Pai SB, Bellamkonda RV, Jeffery ND (2018). Therapeutic efficacy of microtube-embedded chondroitinase ABC in a canine clinical model of spinal cord injury. Brain.

[63] Huang SH, Duan S, Sun T, Wang J, Zhao L, Geng Z, Yan J, Sun HJ, Chen ZY (2011). JIP3 mediates TrkB axonal anterograde transport and enhances BDNF signaling by directly bridging TrkB with kinesin-1. J Neurosci.

[64] Hutson TH, Kathe C, Palmisano I, Bartholdi K, Hervera A, De Virgiliis F, McLachlan E, Zhou L, Kong G, Barraud Q, Danzi MC, Medrano-Fernandez A, Lopez-Atalaya JP, Boutillier AL, Sinha SH, Singh AK, Chaturbedy P, Moon LDF, Kundu TK, Bixby JL, Lemmon VP, Barco A, Courtine G, Di Giovanni S (2019). Cbp-dependent histone acetylation mediates axon regeneration induced by environmental enrichment in rodent spinal cord injury models. Sci Transl Med.

[65] Ibrahim A, Li D, Collins A, Tabakow P, Raisman G, Li Y (2014). Comparison of olfactory bulbar and mucosal cultures in a rat rhizotomy model. Cell Transplant.

[66] Jacobson RD, Virag I, Skene JH (1986). A protein associated with axon growth, GAP-43, is widely distributed and developmentally regulated in rat CNS. J Neurosci.

[67] Jakovcevski I, Djogo N, Holters LS, Szpotowicz E, Schachner M (2013). Transgenic overexpression of the cell adhesion molecule L1 in neurons facilitates recovery after mouse spinal cord injury. Neuroscience.

[68] Kalinski AL, Sachdeva R, Gomes C, Lee SJ, Shah Z, Houle JD, Twiss JL (2015). mRNAs and protein synthetic machinery localize into regenerating spinal cord axons when they are provided a substrate that supports growth. J Neurosci.

[69] Kamber D, Erez H, Spira ME (2009). Local calcium-dependent mechanisms determine whether a cut axonal end assembles a retarded endbulb or competent growth cone. Exp Neurol.

[70] Kappagantula S, Andrews MR, Cheah M, Abad-Rodriguez J, Dotti CG, Fawcett JW (2014). Neu3 sialidase-mediated ganglioside conversion is necessary for axon regeneration and is blocked in CNS axons. J Neurosci.

[71] Koseki H, Donega M, Lam BY, Petrova V, van Erp S, Yeo GS, Kwok JC, Ffrench-Constant C, Eva R, Fawcett JW (2017). Selective rab11 transport and the intrinsic regenerative ability of CNS axons. Elife.

[72] Kreis P, Leondaritis G, Lieberam I, Eickholt BJ (2014). Subcellular targeting and dynamic regulation of PTEN: implications for neuronal cells and neurological disorders. Front Mol Neurosci.

[73] Kumamaru H, Lu P, Rosenzweig ES, Kadoya K, Tuszynski MH (2019). Regenerating corticospinal axons innervate phenotypically appropriate neurons within neural stem cell grafts. Cell Rep.

[74] Lang C, Bradley PM, Jacobi A, Kerschensteiner M, Bareyre FM (2013). STAT3 promotes corticospinal remodelling and functional recovery after spinal cord injury. EMBO Rep.

[75] Lasiecka ZM, Winckler B (2011). Mechanisms of polarized membrane trafficking in neurons—focusing in on endosomes. Mol Cell Neurosci.

[76] Lazo OM, Gonzalez A, Ascano M, Kuruvilla R, Couve A, Bronfman FC (2013). BDNF regulates Rab11-mediated recycling endosome dynamics to induce dendritic branching. J Neurosci.

[77] Lehmann M, Fournier A, Selles-Navarro I, Dergham P, Sebok A, Leclerc N, Tigyi G, McKerracher L (1999). Inactivation of Rho signaling pathway promotes CNS axon regeneration. J Neurosci.

[78] Leibinger M, Andreadaki A, Gobrecht P, Levin E, Diekmann H, Fischer D (2016). Boosting central nervous system axon regeneration by circumventing limitations of natural cytokine signaling. Mol Ther.

[79] Leung KM, van Horck FP, Lin AC, Allison R, Standart N, Holt CE (2006). Asymmetrical beta-actin mRNA translation in growth cones mediates attractive turning to netrin-1. Nat Neurosci.

[80] Li Y, Field PM, Raisman G (1997). Repair of adult rat corticospinal tract by transplants of olfactory ensheathing cells. Science.

[81] Lingor P, Tonges L, Pieper N, Bermel C, Barski E, Planchamp V, Bahr M (2008). ROCK inhibition and CNTF interact on intrinsic signalling pathways and differentially regulate survival and regeneration in retinal ganglion cells. Brain.

[82] Liu K, Lu Y, Lee JK, Samara R, Willenberg R, Sears-Kraxberger I, Tedeschi A, Park KK, Jin D, Cai B, Xu B, Connolly L, Steward O, Zheng B, He Z (2010). PTEN deletion enhances the regenerative ability of adult corticospinal neurons. Nat Neurosci.

[83] Liu Y, Wang X, Li W, Zhang Q, Li Y, Zhang Z, Zhu J, Chen B, Williams PR, Zhang Y, Yu B, Gu X, He Z (2017). A sensitized IGF1 treatment restores corticospinal axon-dependent functions. Neuron.

[84] Lu P, Kadoya K, Tuszynski MH (2014). Axonal growth and connectivity from neural stem cell grafts in models of spinal cord injury. Curr Opin Neurobiol.

[85] Lu P, Wang Y, Graham L, McHale K, Gao M, Wu D, Brock J, Blesch A, Rosenzweig ES, Havton LA, Zheng B, Conner JM, Marsala M, Tuszynski MH (2012). Long-distance growth and connectivity of neural stem cells after severe spinal cord injury. Cell.

[86] Lu P, Woodruff G, Wang Y, Graham L, Hunt M, Wu D, Boehle E, Ahmad R, Poplawski G, Brock J, Goldstein LS, Tuszynski MH (2014). Long-distance axonal growth from human induced pluripotent stem cells after spinal cord injury. Neuron.

[87] Maday S, Twelvetrees AE, Moughamian AJ, Holzbaur EL (2014). Axonal transport: cargo-specific mechanisms of motility and regulation. Neuron.

[88] Mar FM, Simoes AR, Leite S, Morgado MM, Santos TE, Rodrigo IS, Teixeira CA, Misgeld T, Sousa MM (2014). CNS axons globally increase axonal transport after peripheral conditioning. J Neurosci.

[89] Matamoros AJ, Tom VJ, Wu D, Rao Y, Sharp DJ, Baas PW (2019). Knockdown of fidgetin improves regeneration of injured axons by a microtubule-based mechanism. J Neurosci.

[90] Mehta ST, Luo X, Park KK, Bixby JL, Lemmon VP (2016). Hyperactivated Stat3 boosts axon regeneration in the CNS. Exp Neurol.

[91] Miller FD, Tetzlaff W, Bisby MA, Fawcett JW, Milner RJ (1989). Rapid induction of the major embryonic alpha-tubulin mRNA, T alpha 1, during nerve regeneration in adult rats. J Neurosci.

[92] Miyamoto K, Nguyen KT, Allen GE, Jullien J, Kumar D, Otani T, Bradshaw CR, Livesey FJ, Kellis M, Gurdon JB (2018). Chromatin accessibility impacts transcriptional reprogramming in oocytes. Cell Rep.

[93] Montagnac G, Sibarita JB, Loubery S, Daviet L, Romao M, Raposo G, Chavrier P (2009). ARF6 Interacts with JIP4 to control a motor switch mechanism regulating endosome traffic in cytokinesis. Curr Biol.

[94] Moon LDF (2018). Chromatolysis: do injured axons regenerate poorly when ribonucleases attack rough endoplasmic reticulum, ribosomes and RNA?. Dev Neurobiol.

[95] Neumann S, Woolf CJ (1999). Regeneration of dorsal column fibers into and beyond the lesion site following adult spinal cord injury. Neuron.

[96] Nix P, Hammarlund M, Hauth L, Lachnit M, Jorgensen EM, Bastiani M (2014). Axon regeneration genes identified by RNAi screening in *C. elegans*. J Neurosci..

[97] Oblinger MM, Lasek RJ (1988). Axotomy-induced alterations in the synthesis and transport of neurofilaments and microtubules in dorsal root ganglion cells. J Neurosci.

[98] Ogawa Y, Sawamoto K, Miyata T, Miyao S, Watanabe M, Nakamura M, Bregman BS, Koike M, Uchiyama Y, Toyama Y, Okano H (2002). Transplantation of in vitro-expanded fetal neural progenitor cells results in neurogenesis and functional recovery after spinal cord contusion injury in adult rats. J Neurosci Res.

[99] Panayotis N, Karpova A, Kreutz MR, Fainzilber M (2015). Macromolecular transport in synapse to nucleus communication. Trends Neurosci.

[100] Park KK, Liu K, Hu Y, Kanter JL, He Z (2010). PTEN/mTOR and axon regeneration. Exp Neurol.

[101] Pearse DD, Pereira FC, Marcillo AE, Bates ML, Berrocal YA, Filbin MT, Bunge MB (2004). cAMP and Schwann cells promote axonal growth and functional recovery after spinal cord injury. Nat Med.

[102] Piper M, Anderson R, Dwivedy A, Weinl C, van Horck F, Leung KM, Cogill E, Holt C (2006). Signaling mechanisms underlying Slit2-induced collapse of *Xenopus* retinal growth cones. Neuron.

[103] Puttagunta R, Tedeschi A, Soria MG, Hervera A, Lindner R, Rathore KI, Gaub P, Joshi Y, Nguyen T, Schmandke A, Laskowski CJ, Boutillier AL, Bradke F, Di Giovanni S (2014). PCAF-dependent epigenetic changes promote axonal regeneration in the central nervous system. Nat Commun.

[104] Raivich G, Makwana M (2007). The making of successful axonal regeneration: genes, molecules and signal transduction pathways. Brain Res Rev.

[105] Ramer MS, Bishop T, Dockery P, Mobarak MS, O'Leary D, Fraher JP, Priestley JV, McMahon SB (2002). Neurotrophin-3-mediated regeneration and recovery of proprioception following dorsal rhizotomy. Mol Cell Neurosci.

[106] Ramon y Cajal S (1928) Degeneration and regeneration in the nervous system. In: X[1mX[5m*X[0m. English translation 1959. Hafner Press, New York

[107] Rao K, Stone MC, Weiner AT, Gheres KW, Zhou C, Deitcher DL, Levitan ES, Rolls MM (2016). Spastin, atlastin, and ER relocalization are involved in axon but not dendrite regeneration. Mol Biol Cell.

[108] Reier PJ, Bregman BS, Wujek JR (1986). Intraspinal transplantation of embryonic spinal cord tissue in neonatal and adult rats. J Comp Neurol.

[109] Rishal I, Fainzilber M (2014). Axon-soma communication in neuronal injury. Nat Rev Neurosci.

[110] Ritfeld GJ, Nandoe Tewarie RD, Vajn K, Rahiem ST, Hurtado A, Wendell DF, Roos RA, Oudega M (2012). Bone marrow stromal cell-mediated tissue sparing enhances functional repair after spinal cord contusion in adult rats. Cell Transplant.

[111] Romanelli RJ, LeBeau AP, Fulmer CG, Lazzarino DA, Hochberg A, Wood TL (2007). Insulin-like growth factor type-I receptor internalization and recycling mediate the sustained phosphorylation of Akt. J Biol Chem.

[112] Romero MI, Rangappa N, Li L, Lightfoot E, Garry MG, Smith GM (2000). Extensive sprouting of sensory afferents and hyperalgesia induced by conditional expression of nerve growth factor in the adult spinal cord. J Neurosci.

[113] Rosenzweig ES, Brock JH, Lu P, Kumamaru H, Salegio EA, Kadoya K, Weber JL, Liang JJ, Moseanko R, Hawbecker S, Huie JR, Havton LA, Nout-Lomas YS, Ferguson AR, Beattie MS, Bresnahan JC, Tuszynski MH (2018). Restorative effects of human neural stem cell grafts on the primate spinal cord. Nat Med.

[114] Ruschel J, Bradke F (2018). Systemic administration of epothilone D improves functional recovery of walking after rat spinal cord contusion injury. Exp Neurol.

[115] Sahoo PK, Lee SJ, Jaiswal PB, Alber S, Kar AN, Miller-Randolph S, Taylor EE, Smith T, Singh B, Ho TS, Urisman A, Chand S, Pena EA, Burlingame AL, Woolf CJ, Fainzilber M, English AW, Twiss JL (2018). Axonal G3BP1 stress granule protein limits axonal mRNA translation and nerve regeneration. Nat Commun.

[116] Sahoo PK, Smith DS, Perrone-Bizzozero N, Twiss JL (2018). Axonal mRNA transport and translation at a glance. J Cell Sci.

[117] Sasaki M, Radtke C, Tan AM, Zhao P, Hamada H, Houkin K, Honmou O, Kocsis JD (2009). BDNF-hypersecreting human mesenchymal stem cells promote functional recovery, axonal sprouting, and protection of corticospinal neurons after spinal cord injury. J Neurosci.

[118] Schwab ME, Strittmatter SM (2014). Nogo limits neural plasticity and recovery from injury. Curr Opin Neurobiol.

[119] Sekine Y, Lin-Moore A, Chenette DM, Wang X, Jiang Z, Cafferty WB, Hammarlund M, Strittmatter SM (2018). Functional genome-wide screen identifies pathways restricting central nervous system axonal regeneration. Cell Rep.

[120] Sendtner M, Stöckli KA, Thoenen H (1992). Synthesis and localization of ciliary neurotrophic factor in the sciatic nerve of the adult rat after lesion and during regeneration. J Cell Biol.

[121] Shigeoka T, Jung H, Jung J, Turner-Bridger B, Ohk J, Lin JQ, Amieux PS, Holt CE (2016). Dynamic axonal translation in developing and mature visual circuits. Cell.

[122] Sinclair SR, Fawcett JW, Dunnett SB (1999). Surviving dopamine cells in nigral grafts differentiate prior to implantation. Eur J Neurosci.

[123] Sorg BA, Berretta S, Blacktop JM, Fawcett JW, Kitagawa H, Kwok JC, Miquel M (2016). Casting a wide net: role of perineuronal nets in neural plasticity. J Neurosci.

[124] Stenevi U, Bjorklund A, Svendgaard N-A (1976). Transplantation of central and peripheral monoamine neurons to the adult rat brain: techniques and conditions for survival. Brain Res.

[125] Subang MC, Richardson PM (2001). Synthesis of leukemia inhibitory factor in injured peripheral nerves and their cells. Brain Res.

[126] Sykova E, Homola A, Mazanec R, Lachmann H, Konradova SL, Kobylka P, Padr R, Neuwirth J, Komrska V, Vavra V, Stulik J, Bojar M (2006). Autologous bone marrow transplantation in patients with subacute and chronic spinal cord injury. Cell Transplant.

[127] Tabakow P, Raisman G, Fortuna W, Czyz M, Huber J, Li D, Szewczyk P, Okurowski S, Miedzybrodzki R, Czapiga B, Salomon B, Halon A, Li Y, Lipiec J, Kulczyk A, Jarmundowicz W (2014). Functional regeneration of supraspinal connections in a patient with transected spinal cord following transplantation of bulbar olfactory ensheathing cells with peripheral nerve bridging. Cell Transplant.

[128] Tan CL, Kwok JC, Patani R, Ffrench-Constant C, Chandran S, Fawcett JW (2011). Integrin activation promotes axon growth on inhibitory chondroitin sulfate proteoglycans by enhancing integrin signaling. J Neurosci.

[129] Tedeschi A, Dupraz S, Laskowski CJ, Xue J, Ulas T, Beyer M, Schultze JL, Bradke F (2016). The calcium channel subunit alpha2delta2 suppresses axon regeneration in the adult CNS. Neuron.

[130] Tetzlaff W, Kobayashi NR, Giehl KMG, Tsui BJ, Cassar SL, Bedard AM (1994). Response of rubrospinal and corticospinal neurons to injury and neurotrophins. Prog Brain Res.

[131] Thompson A, Berry M, Logan A, Ahmed Z (2019). Activation of the BMP4/Smad1 pathway promotes retinal ganglion cell survival and axon regeneration. Invest Ophthalmol Vis Sci.

[132] Tom VJ, Steinmetz MP, Miller JH, Doller CM, Silver J (2004). Studies on the development and behavior of the dystrophic growth cone, the hallmark of regeneration failure, in an in vitro model of the glial scar and after spinal cord injury. J Neurosci.

[133] Trakhtenberg EF, Li Y, Feng Q, Tso J, Rosenberg PA, Goldberg JL, Benowitz LI (2018). Zinc chelation and Klf9 knockdown cooperatively promote axon regeneration after optic nerve injury. Exp Neurol.

[134] Tran AP, Warren PM, Silver J (2018). The biology of regeneration failure and success after spinal cord injury. Physiol Rev.

[135] Twiss JL, Kalinski AL, Sachdeva R, Houle JD (2016). Intra-axonal protein synthesis - a new target for neural repair?. Neural Regen Res.

[136] Urdzikova L, Jendelova P, Glogarova K, Burian M, Hajek M, Sykova E (2006). Transplantation of bone marrow stem cells as well as mobilization by granulocyte-colony stimulating factor promotes recovery after spinal cord injury in rats. J Neurotrauma.

[137] Vecino E, Heller JP, Veiga-Crespo P, Martin KR, Fawcett JW (2015). Influence of extracellular matrix components on the expression of integrins and regeneration of adult retinal ganglion cells. PLoS ONE.

[138] Venkatesh I, Mehra V, Wang Z, Califf B, Blackmore MG (2018). Developmental chromatin restriction of pro-growth gene networks acts as an epigenetic barrier to axon regeneration in cortical neurons. Dev Neurobiol.

[139] Verma P, Chierzi S, Codd AM, Campbell DS, Meyer RL, Holt CE, Fawcett JW (2005). Axonal protein synthesis and degradation are necessary for efficient growth cone regeneration. J Neurosci.

[140] Vogelaar CF, Gervasi NM, Gumy LF, Story DJ, Raha-Chowdhury R, Leung KM, Holt CE, Fawcett JW (2009). Axonal mRNAs: Characterisation and role in the growth and regeneration of dorsal root ganglion axons and growth cones. Mol Cell Neurosci.

[141] Wang X, Yigitkanli K, Kim CY, Sekine-Komo T, Wirak D, Frieden E, Bhargava A, Maynard G, Cafferty WB, Strittmatter SM (2014). Human NgR-Fc decoy protein via lumbar intrathecal bolus administration enhances recovery from rat spinal cord contusion. J Neurotrauma.

[142] Watkins TA, Wang B, Huntwork-Rodriguez S, Yang J, Jiang Z, Eastham-Anderson J, Modrusan Z, Kaminker JS, Tessier-Lavigne M, Lewcock JW (2013). DLK initiates a transcriptional program that couples apoptotic and regenerative responses to axonal injury. Proc Natl Acad Sci USA.

[143] Wictorin K, Brundin P, Gustavii B, Lindvall O, Björklund A (1990). Reformation of long axon pathways in adult rat central nervous system by human forebrain neuroblasts. Nature.

[144] Yan D, Wu Z, Chisholm AD, Jin Y (2009). The DLK-1 kinase promotes mRNA stability and local translation in *C. elegans* synapses and axon regeneration. Cell.

[145] Yang LJ, Lorenzini I, Vajn K, Mountney A, Schramm LP, Schnaar RL (2006). Sialidase enhances spinal axon outgrowth in vivo. Proc Natl Acad Sci USA.

[146] Zhou CF, Raisman G, Morris RJ (1985). Specific patterns of fibre outgrowth from transplants to host mice hippocampi, shown immunohistochemically by the use of allelic forms of Thy-1. Neuroscience.

